# Persistent left superior vena cava with absent right superior vena cava: a case report and review of the literature

**Published:** 2010-06

**Authors:** ÖZGÜL UÇAR, HÜLYA ÇİÇEKÇİOĞLU, İBRAHİM KOCAOĞLU, SİNAN AYDOĞDU, LALE PAŞAOĞLU, MURAT VURAL

**Affiliations:** Department of Cardiology, Ankara Numune Education and Research Hospital, Sihhiye, Ankara, Turkey; Department of Cardiology, Ankara Numune Education and Research Hospital, Sihhiye, Ankara, Turkey; Department of Cardiology, Ankara Numune Education and Research Hospital, Sihhiye, Ankara, Turkey; Department of Cardiology, Ankara Numune Education and Research Hospital, Sihhiye, Ankara, Turkey; Department of Radiology, Ankara Numune Education and Research Hospital, Sihhiye, Ankara, Turkey; Department of Radiology, Ankara Numune Education and Research Hospital, Sihhiye, Ankara, Turkey

**Keywords:** echocardiography, vascular malformation, superior vena cava

## Abstract

We report on a rare case of persistent left superior vena cava (PLSVC) with absent right superior vena cava (RSVC), an anomaly that is also known as isolated PLSVC. This venous malformation was identified incidentally in a 30-year-old woman during thoracic multi-detector computed tomography (MDCT), which was performed with the suspicion of intra-thoracic malignancy.

On thoracic MDCT, the RSVC was absent. A bridging vein drained the right jugular and right subclavian veins and joined the left brachiocephalic vein in order to form the PLSVC, which descended on the left side of the mediastinum and drained into the right atrium (RA) via a dilated coronary sinus (CS). The patient was referred to the cardiology department for further evaluation. Transthoracic echocardiography revealed a dilated CS, and agitated saline injected from the left or right arms revealed opacification of the CS before the RA. The patient had no additional cardiac abnormality.

Isolated PLSVC is usually asymptomatic but it can pose difficulties with central venous access, pacemaker implantation and cardiothoracic surgery. This condition is also associated with an increased incidence of congenital heart disease, arrhythmias and conduction disturbances. A wide spectrum of clinicians should be aware of this anomaly, its variations and possible complications.

## Introduction

Persistent left superior vena cava (PLSVC) is the most common congenital malformation of the thoracic venous system and it affects about 0.3 to 0.5% of the general population.^1^ This incidence increases 10-fold in patients with cardiac malformations. 2 PLSVC with an absent right superior vena cava (RSVC), which is also referred to as isolated PLSVC, is very uncommon, occurring in 0.07 to 0.13% of patients who have congenital heart defects with viscero-atrial situs solitus. Nearly half of the patients with isolated PLSVC have other cardiac malformations, such as atrial septal defect, endocardial cushion defect or tetralogy of Fallot.3 In this case report we present a patient with isolated PLSVC with no other cardiac abnormalities, who was diagnosed incidentally during thoracic computed tomography.

## Case report

**Fig. 1.**
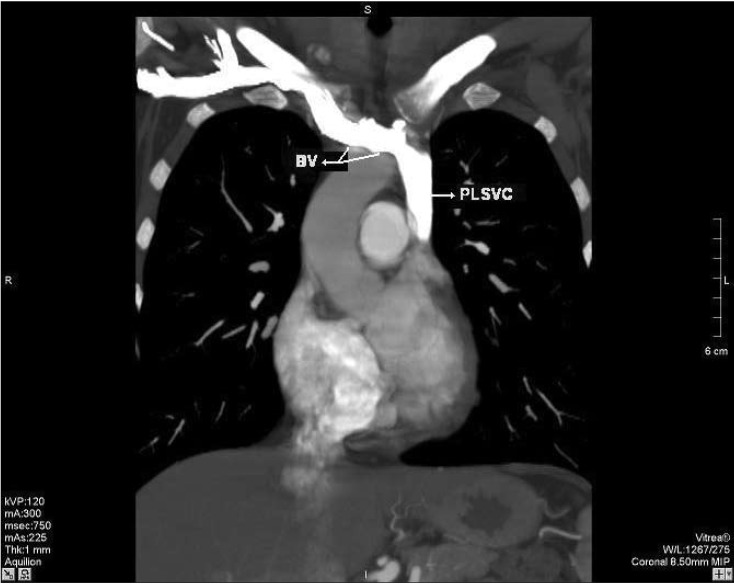
Multiplanar reformatted image demonstrates that the right superior vena cava is absent and a bridging vein (BV) drains the right jugular and subclavian veins, which then join with the left brachiocephalic vein to form the persistent left superior vena cava (PLSVC).

**Fig. 2.**
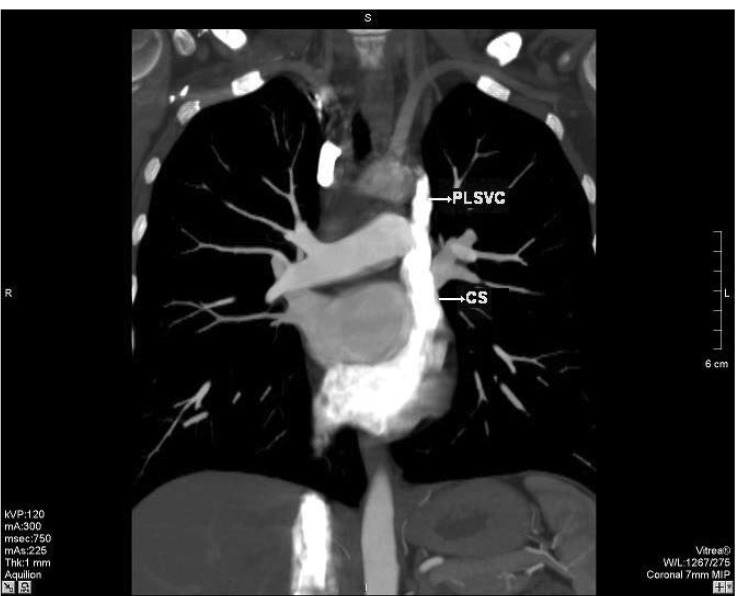
Multiplanar reformatted image reveals the persistent left superior vena cava (PLSV C) draining into a dilated coronary sinus (CS).

**Fig. 3.**
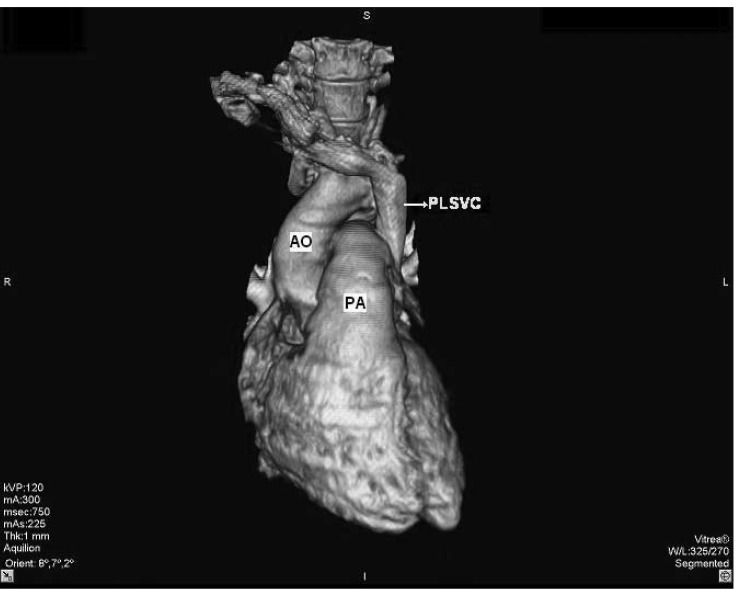
Volume-rendered, three-dimensional reconstruction of contrast-enhanced multi-detector computed tomographic image shows the persistent left superior vena cava (PLSV C) descending on the left side of the thorax (AO: aorta, PA: pulmonary artery).

A 30-year-old Caucasian woman went on contrast-enhanced thoracic 16-row multi-detector computed tomography (MDCT) for the suspicion of intra-thoracic malignancy. MDCT showed a bridging vein draining the right jugular and right subclavian veins; it joined the left brachiocephalic vein and formed the PLSVC, which descended at the left side of the mediastinum leftward of the pulmonary artery and left atrium (LA) before draining into the right atrium (RA) via a dilated coronary sinus (CS) (Figs 1–3). The RSVC was absent and the PLSVC carried all venous blood from the head, neck and upper extremities. There was no other pathological finding. The visceral organs were normally positioned. The patient was referred to the cardiology department for further research for a cardiac abnormality.

**Fig. 4.**
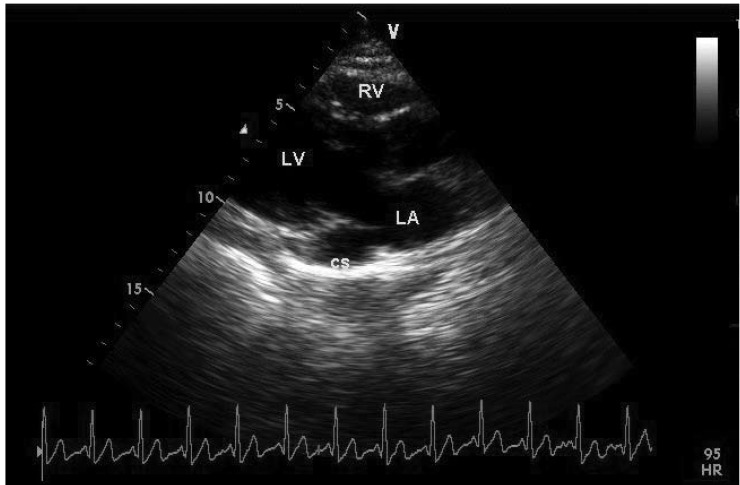
Dilated coronary sinus (CS) on transthoracic echocardiography, parasternal long-axis view (LA : left atrium, LV : left ventricle, RV : right ventricle).

On examination, the general appearance of the patient was good. Her blood pressure was 110/80 mmHg and pulse rate was 85 beats/min. Cardiac auscultation and electrocardiogram (ECG) were normal. Transthoracic echocardiography (TTE) revealed a dilated CS of 31 × 19 mm (Fig. 4). There was no evidence of valvular heart disease and the diameters of the heart chambers were within normal limits. The left ventricular ejection fraction was 68% and the estimated peak systolic pulmonary artery pressure was in the normal range.

The inter-atrial and inter-ventricular septum were both intact. All four pulmonary veins drained into the LA. The thickness of the pericardium was normal with no effusion. An agitated saline injection was made from the left antecubital vein. The contrast first appeared in the CS, then in the RA. The injection was repeated from the right antecubital vein, which also demonstrated appearance of the contrast first in the CS and afterwards in the RA. The diagnosis was confirmed and no additional cardiac abnormality was detected.

## Discussion

PLSVC with absent RSVC (isolated PLSVC) is a very rare venous malformation. During normal foetal development, the left-sided anterior venous cardinal system regresses, leaving the CS and the ligament of Marshall. Failure of the closure of the left anterior cardinal vein results in PLSVC.^4^ In general, PLSVC is associated with RSVC and drains into the RA via a dilated CS. When developmental arrest occurs at an earlier stage, the CS is absent and the PLSVC drains into the LA. Either isolated or associated with RSVC, this venous malformation itself causes no haemodynamic disturbance and is usually diagnosed incidentally. 5-7

However, it has several clinical implications. A PLSVC can cause problems during central venous catheterisation (access to the CS can cause hypotension, angina, perforation of the heart, tamponade and arrest),^8^ pacemaker implantation (due to the circuitous path taken by the electrode, it can be difficult to obtain a stable electrode position and sustained capture),^9^ or cardiopulmonary bypass (isolated PLSVC impairs the use of retrograde cardioplegia).

In addition, a higher incidence of arrhythmias and conduction system abnormalities has been described in patients with PLSVC. There are two proposed mechanisms for this association: a dilated CS stretches the atrioventricular nodal tissue, which prepares a substrate for re-entrant tachycardias; or, the early conduction tissue has close proximity to the cardinal venous tissue and this leads to sinus node dysfunction. Lenox et al. found sino-atrial node abnormalities in some patients with absent RSVC and this condition may predispose to sick sinus syndrome.^10^-^12^

In 10% of patients, a PLSVC may drain into the LA either directly or via an unroofed CS. This creates a right-to-left shunt and the risk of paradoxical embolism is markedly increased. In addition, drugs directly enter the systemic circulation when they are applied from the left brachiocephalic vein.

A final clinical implication of PLSVC (especially when isolated) is a high incidence of accompanying congenital heart defects, for example ventricular septal defect, atrial septal defect, endocardial cushion defect or tetralogy of Fallot.^13^,^13^ Therefore associated congenital heart disease should be meticulously searched for.

When PLSVC is present, the ECG often shows an abnormal P-wave axis and a normal or shortened PR interval. A geometric change in the LA may be a possible mechanism for the left-axis deviation of the P wave.^14^ On chest X-ray, a crescent-shaped shadow of the PLSVC can be seen at the aortic knob or left upper mediastinum. After insertion of a pulmonary artery catheter into the left subclavian or jugular vein, a control chest X-ray gives the false appearance that the catheter has passed through the vessel. The diagnosis can be confirmed by TTE, transoesophageal echocardiography (TEE), venous angiography, computed tomography (CT) or magnetic resonance imaging (MRI).

On two-dimensional B-mode TTE, the characteristic finding is a dilated CS on parasternal long-axis view. The normal diameter of the CS is smaller than 1 cm and in the case of isolated PLSVC, severely increased flow can cause a truly giant CS.^15^,^16^ Other causes of dilated CS are: increased RA pressure, an anomalous systemic or pulmonary venous connection or a fistulous connection with the coronary arteries.^17^

The next step in the echocardiographic evaluation should be contrast application with agitated saline. In normal individuals, agitated saline injection from the left or right antecubital vein results in opacification of the RA. In isolated PLSVC, as in our case, contrast given from the left or right arm opacifies the CS. When PLSVC is associated with an unroofed CS, contrast injection from either arm results in opacification of the LA. If RSVC accompanies the PLSVC, contrast given from the left arm first appears in the CS, whereas contrast given from the right arm first appears in the RA (Table 1).

**Table 1:** ECHOCARDIOGRAPHIC DIAGNOSIS OF PLSVC AND ITS POSSIBLE VARIATIONS WITH CONTRAST APPLICATION

**	*Normal*	PLSVC without RSVC*	*PLSVC with RSVC*	*PLSVC with unroofed CS*
Contrast from the left arm	RA	CS→RA	CS→RA	LA
Contrast from the right arm	RA	CS→RA	RA	LA

PLSVC: persistent left superior vena cava, RSVC: right superior vena cava, RA: right atrium, LA: left atrium, CS: coronary sinus. * isolated PLSVC.

On TEE, the anomalous PLSVC and absence of RSVC can be well visualised. In mid-oesophageal views, the PLSVC can be seen near to the left atrial appendage and left upper pulmonary vein. In the bicaval view, the absence of RSVC can be demonstrated. Other techniques (venous angiography, CT, MRI) directly visualise the venous anatomy and confirm the diagnosis.

In the absence of an RSVC, central venous access should be made from the femoral vein in patients with PLSVC. During right-sided open-heart surgical procedures, a PLSVC has to be drained by inserting a separate cannula into it. If the PLSVC drains into the LA and creates a large right-to-left shunt, surgical correction should be made. Again, central venous access via the femoral vein is a safer choice in this variation. When implanting permanent pacemakers, the left subclavian vein is preferred, as lead manipulation is easier. There is an acute angle between the CS ostium and the tricuspid valve, therefore the lead should be looped in the RA in order to enter the right ventricle.^18^ Handshaped stylets and active fixation leads are also helpful to overcome overcome technical difficulties.^19^

Finally, a wide spectrum of clinicians (radiologists, sonographers, intervenists, intensivists, anaesthesiologists, cardiothoracic surgeons) should be aware of PLSVC and its variations in order to avoid possible complications.
